# Accuracy of Intraoral Digital Impressions for Whole Upper Jaws, Including Full Dentitions and Palatal Soft Tissues

**DOI:** 10.1371/journal.pone.0158800

**Published:** 2016-07-06

**Authors:** Ning Gan, Yaoyang Xiong, Ting Jiao

**Affiliations:** Department of Prosthodontics, Ninth People's Hospital, affiliated to Shanghai Jiao Tong University School of Medicine, Shanghai Key Laboratory of Stomatology, Shanghai, PR China; Second University of Naples, ITALY

## Abstract

Intraoral digital impressions have been stated to meet the clinical requirements for some teeth-supported restorations, though fewer evidences were proposed for larger scanning range. The aim of this study was to compare the accuracy (trueness and precision) of intraoral digital impressions for whole upper jaws, including the full dentitions and palatal soft tissues, as well as to determine the effect of different palatal vault height or arch width on accuracy of intraoral digital impressions. Thirty-two volunteers were divided into three groups according to the palatal vault height or arch width. Each volunteer received three scans with TRIOS intraoral scanner and one conventional impression of whole upper jaw. Three-dimensional (3D) images digitized from conventional gypsum casts by a laboratory scanner were chose as the reference models. All datasets were imported to a specific software program for 3D analysis by "best fit alignment" and "3D compare" process. Color-coded deviation maps showed qualitative visualization of the deviations. For the digital impressions for palatal soft tissues, trueness was (130.54±33.95)μm and precision was (55.26±11.21)μm. For the digital impressions for upper full dentitions, trueness was (80.01±17.78)μm and precision was (59.52±11.29)μm. Larger deviations were found between intraoral digital impressions and conventional impressions in the areas of palatal soft tissues than that in the areas of full dentitions (p<0.001). Precision of digital impressions for palatal soft tissues was slightly better than that for full dentitions (p = 0.049). There was no significant effect of palatal vault height on accuracy of digital impressions for palatal soft tissues (p>0.05), but arch width was found to have a significant effect on precision of intraoral digital impressions for full dentitions (p = 0.016). A linear correlation was found between arch width and precision of digital impressions for whole upper jaws (r = 0.326, p = 0.034 for palatal soft tissues and r = 0.485, p = 0.002 for full dentitions). It was feasible to use the intraoral scanner to obtain digital impressions for whole upper jaws. Wider dental arch contributed to lower precision of an intraoral digital impression. It should be confirmed in further studies that whether accuracy of digital impressions for whole upper jaws is clinically acceptable.

## Introduction

Digital impressions and scanning systems were introduced in dentistry in the mid 1980s[1]. As the initial step of dental CAD/CAM (computer aided designed/computer aided manufactured) techniques, digital impression is increasingly applied in single crowns[2, 3], multi-unit fixed dental prostheses (FDPs), and has expanded in recent years in the field of oral implants[4, 5], complete denture prosthodontics[6] and obturator prostheses[7]. There are two ways to create a digital impression: direct intraoral scanning or indirect extraoral scanning gypsum casts[5]. An extraoral optical scanner can allow a fast and high-resolution data acquisition with the accuracy of 5–10μm[8, 9], while the accuracy of intraoral scanning is stated to be 50μm[9]. A direct intraoral scanning is truly free of a physical impression so that it is able to get rid of the errors derived from the distortion of elastomeric impressions, disproportionate water/powder ratio of dental plaster and unsuitable storage conditions of physical impressions or gypsum casts[10]. Some researchers have demonstrated that veneers, single crowns and FDPs manufactured from direct intraoral scanning data delivered equivalent or even better marginal and internal fit compared with those fabricated from conventional impressions[1–3, 11–14], which means that accuracy of intraoral digital impressions is able to meet the clinical requirements for teeth-supported restorations of short units.

Comparing much progress of CAD/CAM techniques for FDPs with intraoral digital impressions, development for removable partial dentures (RPDs) is relatively slow. It is more complicated because impressions for RPDs may not only cover a wider range of dentitions but also soft tissues that touched by major or minor connectors. It was reported that the flexible oral mucosa and smooth-surface textures covered by saliva might be the challenges when taking the intraoral digital impression for RPDs or complete dentures[15, 16]. To date, there are few reports focused on the feasibility and accuracy of intraoral digital impressions for whole jaws, especially for the part of soft tissues. Most studies mentioned drew conclusions based on standard models or one single patient, yet few took the influence of difference in individual anatomic forms, such as different palatal vault height and arch width, on the accuracy of digital impression into consideration.

The objective of this in vivo study was to compare accuracy of intraoral digital impressions for palatal soft tissues and full upper dentitions, as well as to determine the effect of different palatal vault height or arch width on accuracy of intraoral digital impressions. The null hypothesis states that: (1) it is feasible to use the intraoral scanner to obtain digital impressions for whole upper jaws; (2) the palatal vault height or arch width do not influence the accuracy of intraoral digital impressions.

## Materials and Methods

### Participants

The study was approved by the Independent Ethics Committee of Shanghai Ninth People's Hospital affiliated to Shanghai Jiao Tong University, School of Medicine (Application No. [2014]81). It was conducted in accordance with the Declaration of Helsinki. All of the volunteers who understood the study and were willing to participate in signed the consent form and were recruited for the study.

**Inclusion criteria**
- Volunteers from Shanghai Jiao Tong University School of Medicine;
- Aged at least eighteen years;
- Good oral hygiene;
- Complete maxillary dental arch except the missing third molar;
- Intact hard and soft tissues, including treated teeth decay and healed teeth extraction socket;

**Exclusion criteria**
- Undergoing orthodontic treatment;
- With metal crowns and any other metal materials on teeth;
- With soft tissue lesions and postoperative scars on the palate;
- With oral implants;
- Advanced periodontitis affecting gingival recession;
- Obvious teeth mobility (mobility degree higher than 1);
- Obvious dentition malalignment (malalignment degree higher than 1).

### 3D datasets generation

Every volunteer’s whole upper jaw, including the full dentition and palatal soft tissues, was captured digitally with an intraoral scanner (TRIOS POD, 3 Shape, Copenhagen, Denmark). The scan process was conducted following the manufacturer’s guidelines, before which saliva on volunteer’s palate and teeth was tried to be removed by cotton rolls and air syringe, and buccal or labial mucosa were pulled by mouth mirrors to avoid the negative effects of intraoral conditions as much as possible. Scanning started with the second molar in the first quadrant and ended at the second molar in the second quadrant. Each tooth was scanned from occlusal surface then followed a slow zigzag scanning of the dentition. Scanning of the palatal soft tissues started with the palatal sides of upper central incisors, and moved in a zigzag manner as well until reaching the level of the distal end of the second molars. Before completing the whole scan, missed areas were rescanned and irrelevant areas were removed by trim tool in scanning software. Small mismatches were erased and rescanned. Digital images with large mismatches, which could not be covered by rescanned, were eliminated and a new digital image would be captured from the beginning. Unbroken and smooth digital images were defined as images of good quality. The scanning time and the number of scanning pictures were also recorded in every image acquisition. This process was then repeated two times, so every volunteer had three digital impressions (n = 3 per volunteer, named DI_1_, DI_2_ and DI_3_). All scans were performed by a well-trained dentist. All the DI data were submitted to the laboratory authorized by 3 Shape Corporation, then exported and converted to the Standard Tessellation Language (STL) file format by using a model analysis software (Ortho Analyzer, 3 Shape, Copenhagen, Denmark). An STL file format was compatible with and able to be imported into most 3D model processing softwares.

**Fig 1:**
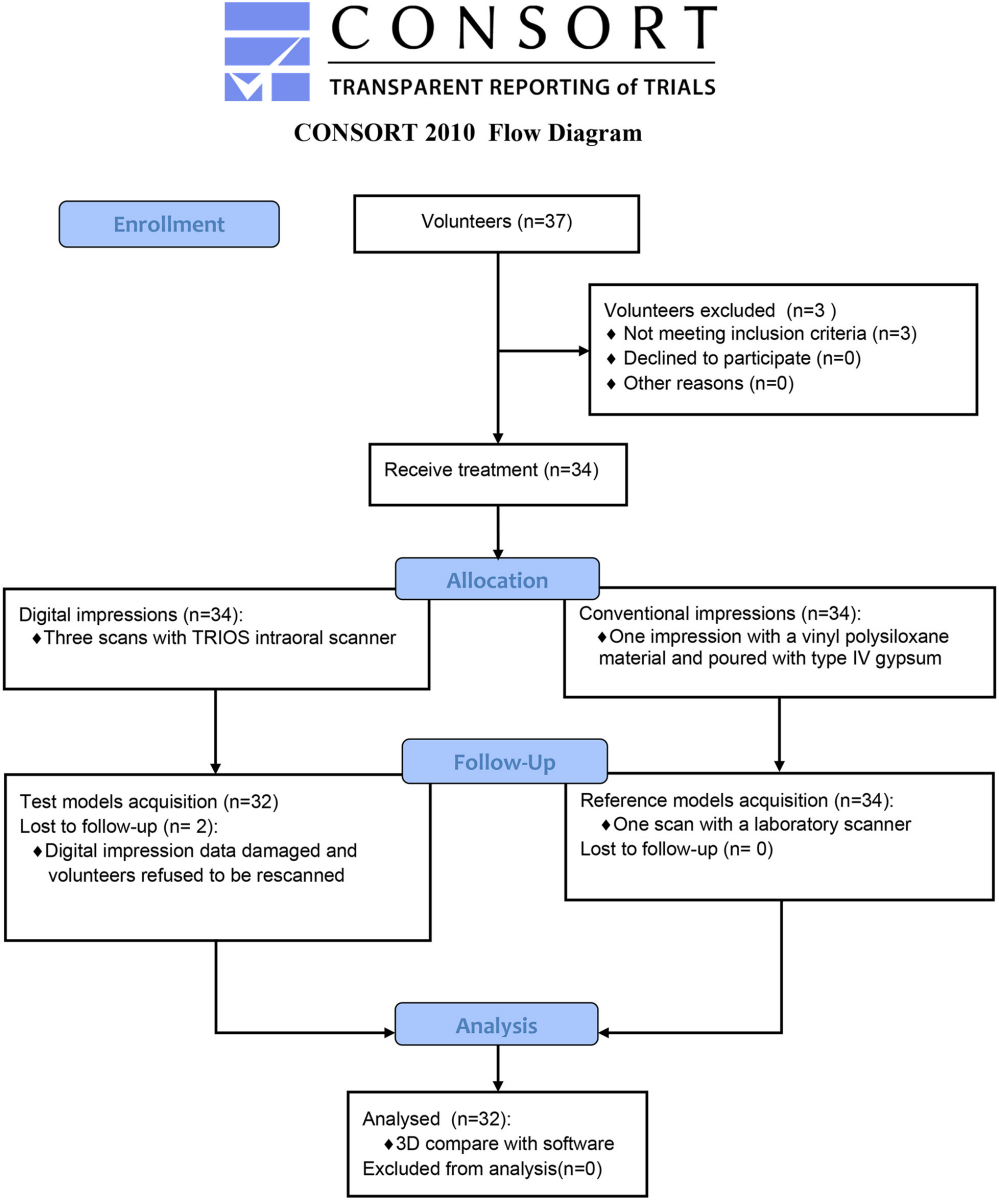
Flow chart.

A volunteer’s conventional impression was obtained right after the completion of intraoral digital scanning. After the best fitting trays selected and the adhesive applied (Tray Adhesive, DMG, Hamburg, Germany), conventional impression procedure was performed with a vinyl polysiloxane material (Honigum Putty/Light, DMG, Hamburg, Germany) in a two-step putty-wash method following the manufacturer’s instruction. Then the conventional impression was poured with scannable type IV gypsum (uni-base 300, Dentona, Germany) and stored at a room temperature of 21–23°C. All the operations were performed at the department of prosthodontics, Ninth People's Hospital by the same dentist mentioned above. It was not suggested to directly scan elastomeric impressions because shadowing effects will limit the use of optical scanner in the data acquisition of cavities or negative moulds[10], and then affect the integrity of final virtual models. Digitized each cast once by a laboratory scanner (D500 3D scanner, 3 Shape, Copenhagen, Denmark) after storage of at least 96 hours until the expansion of gypsum was complete[5, 17]. These virtual 3D images (named CIs) were converted into STL file format and considered as volunteers’ gold standard models. The whole enrollment and allocation of volunteers are shown in Fig 1.

### Measurement of arch width and palatal vault height

**Fig 2:**
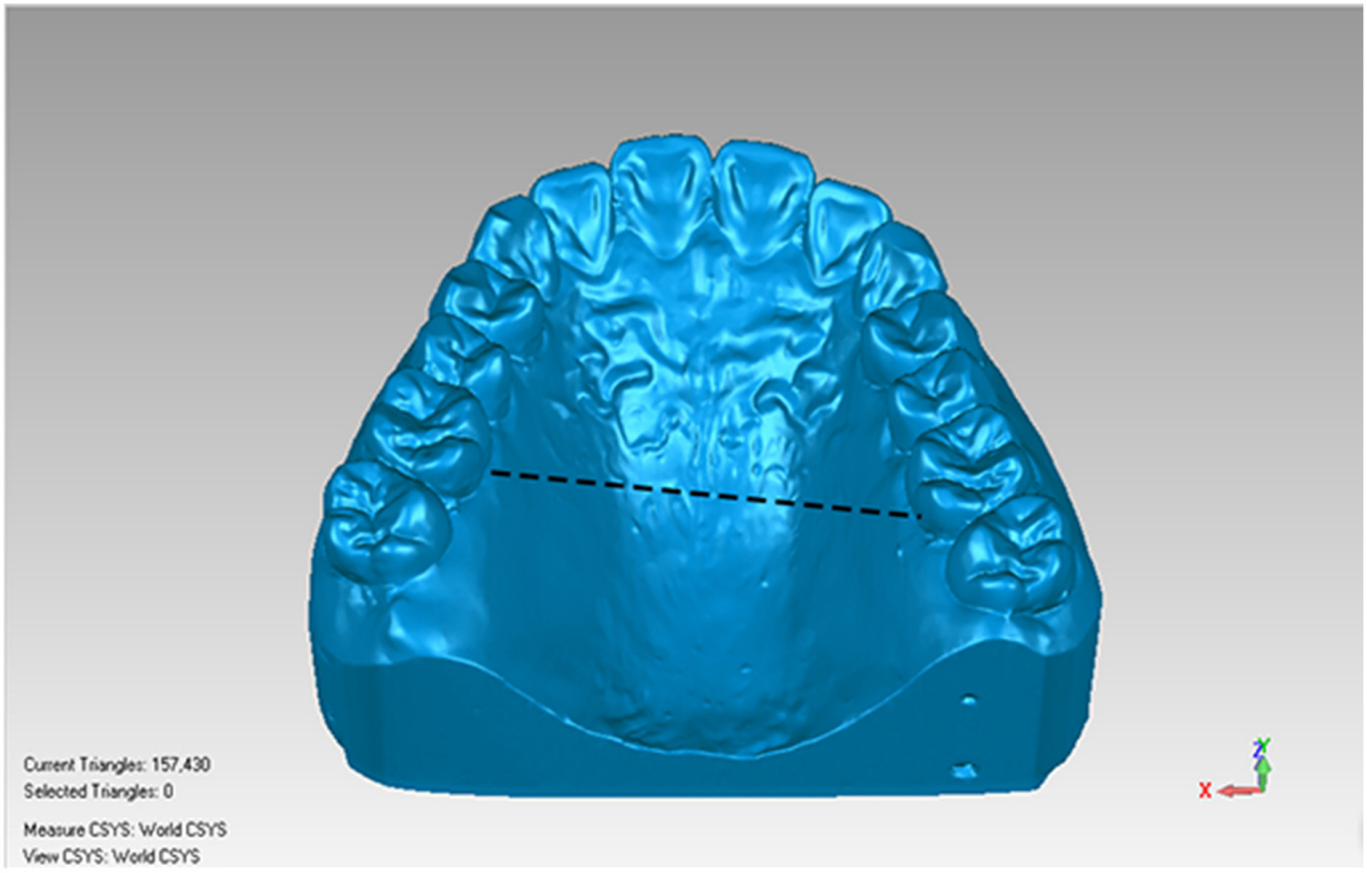
Measurement method of arch width.

**Fig 3:**
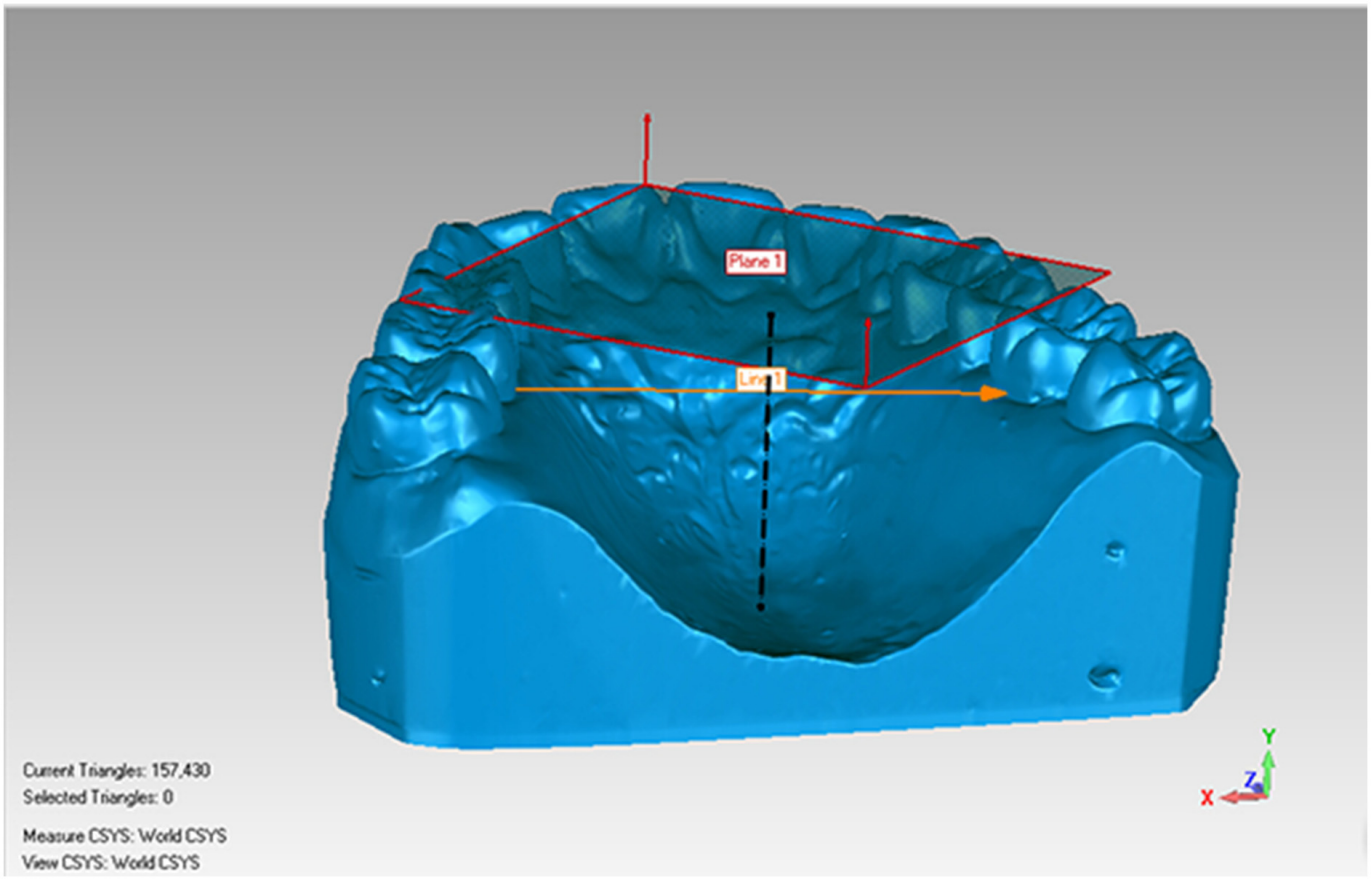
Measurement method of palatal vault height.

Reference models (CIs) were measured using the tool of distance measurement in the reverse engineering software (Geomagic Qualify 12; Geomagic; Morrisville, NC, USA). The arch width was defined as the distance in a straight line between the mesiodistal center of the palatal gingival margin of the left and right first molars. The palatal vault height was defined as the vertical distance from the occlusal plane to the median line of the palate in the position connecting the mesiodistal center of the left and right first molars [18], (Figs 2 and 3). Each volunteer’s arch width and palatal vault height were measured three times, and a mean value was calculated. The volunteers were divided into three arch width groups (Group Narrow, Medium and Wide) and three palatal vault height groups (Group Low, Medium and High) with the reference to Takuya Takanashi’s data[18].

### Analysis of trueness and precision

All STL datasets were imported into Geomagic Studio 12 (Geomagic; Morrisville, NC, USA) for trimming the borders. To make the superimposition more precise, irrelevant areas such as the field outside the distal end of the second molars and soft tissues beyond the buccal side of dentitions were removed after a primary alignment between two surfaces of DI models. Thus, all DI models of each volunteer were cut with the common cutting planes to create equal borders and obtain a same field of interest. For accurate calculation, palatal soft tissues were selected from the whole upper jaw by “Trim with curve” function and analyzed independently from the upper dentitions later on. The STL datasets from the digital impressions and the conventional impressions were imported into Geomagic Qualify 12 again for overall 3D compare. All compares were made by utilization of a repeated best fit algorithm which the test model would be aligned to the reference model automatically in three dimensions. After 3D compares were finished, color-coded deviation maps were generated to show the differences between two aligned models and deviation information, such as average positive deviation, average negative deviation and standard deviation, was presented simultaneously.

Accuracy is described by trueness and precision (ISO 5725–1)[5, 19]. In this study, trueness is defined as the comparison between digital impression served as test model and a conventional impression served as reference model of the same volunteer (that is, DI_1_, DI_2_ and DI_3_ compared with CI respectively; 3 pairs per volunteer). Precision is defined as the comparison between repeated digital scanning models obtained from one volunteer (that is, DI_1_ compared with DI_2_, DI_2_ compared with DI_3_ and DI_3_ compared with DI_1_; 3 pairs per volunteer)[15, 20]. Following the 3D compare of every pairs, deviation information expressed as mean absolute deviation ($\frac{\left|\text{average positive deviation}\right|+\left|\text{average negative deviation}\right|}{2}$) accounting for trueness and standard deviation accounting for precision[5, 10]. The mean deviations of each volunteer were calculated.

### Statistical analysis

The statistical analysis was done with IBM SPSS Statistics Version 21 (IBM SPSS, Chicago, IL, USA). For each group classification, the mean value, the standard deviation (S.D.), the minimum and the maximum were calculated. Statistical analysis was carried out using the Kolmogorov—Smirnov test for normal distribution and Levene’s test for homogeneity of variance. Paired two sample Student’s t-test was used to analyze the differences between accuracy of digital impressions for palatal soft tissues and full dentitions. ANOVA was used to analyze the effect of three different arch width or palatal vault height on accuracy of digital impressions and LSD test was used for further comparison in pairs. Pearson Correlation Coefficient was used to analyze the linear relationship between arch width or palatal vault height and accuracy of digital impressions. We set statistical significance at p < 0.05.

In this study, α = 0.05, β = 0.1, and 1-β = 0.9. The clinically significant level was when the power of the statistical test was larger than 0.9. To determine the sample size, prior to this study we conducted preliminary experiments to calculate the sample size using the following equation[21]:

$$\text{n}=\Psi^{2}(\Sigma {\text{s}}_{\text{i}}{}^{2}/\text{k)/[}\Sigma {\left({\bar{\text{x}}}_{\text{i}}-\bar{\text{x}}\right)}^{2}/(\text{k}-1)]$$ (1)

where k means the number of the groups. s_i_ means the standard deviation of the “i” group. ${\bar{\text{x}}}_{\text{i}}$ means the average value of the “i” group and $\bar{\text{x}}$ means the total average value of the three groups. In this study, k = 3 and Ψ_0.05,0.1,2,∞_ = 2.52. We calculated that the minimum sample size for each arch width group should be 6 volunteers.

## Results

### Volunteer Information

The whole data collection procedure was performed across from January 2015 to January 2016. Thirty-seven adult volunteers participated; a total of thirty-two volunteers meeting the inclusion criteria and obtained informed consent were recruited for this study. 14 (43.75%) volunteers were men and 18 (56.25%) volunteers were women, who aged from twenty-one to forty-one years and the average age was (27.0±5.1) years old. The completed CONSORT flowchart of participants is shown in Fig 1. The classifications of arch width and palatal vault height are shown in Tables 1 and 2. The average scanning time of 32 volunteers was (4mins58secs±1mins17secs) and the average scanning pictures was (835±148), including rescan time and rescan pictures. Two-step putty-wash impression procedures were conducted following the manufacturer’s instruction with four-handed technique. The time for the impression in each volunteer’ mouth was about 7mins30secs (4mins for preliminary impression and 3mins30secs for final impression), exclusive of the preparation time (time for tray selection, adhesive application, spillway carving and so on).

**Table 1:** Classification of Arch Width.

Group	Male (N)^a^	Female (N)^b^	Mean(mm) ^c^	S.D.^d^ (mm)	Minimum(mm)	Maximum(mm)
**Narrow**	2	8	34.18	1.35	31.33	36.21
**Medium**	4	8	38.66	0.96	36.95	39.97
**Wide**	8	2	41.12	0.86	40.16	43.15
**Total**	14	18	38.03	3.01	31.33	43.15

^a^“Male(N)” means the number of male volunteers;

^b^“Female(N)” means the number of female volunteers;

^c^“mm” means millimeter;

^d^ “S.D.” means standard deviations.

**Table 2:** Classification of Palatal Vault Height.

Group	Male (N)^a^	Female (N)^b^	Mean(mm) ^c^	S.D.^d^ (mm)	Minimum(mm)	Maximum(mm)
**Low**	3	6	16.91	0.70	15.56	17.72
**Medium**	5	9	19.27	0.87	18.08	20.95
**High**	6	3	22.85	1.61	21.40	26.66
**Total**	14	18	19.61	2.51	15.56	26.66

^a^“Male(N)” means the number of male volunteers;

^b^“Female(N)” means the number of female volunteers;

^c^“mm” means millimeter;

^d^ “S.D.” means standard deviations.

### Difference between Accuracy of Digital Impressions for Palatal Soft Tissues and Full Dentitions

**Fig 4:**
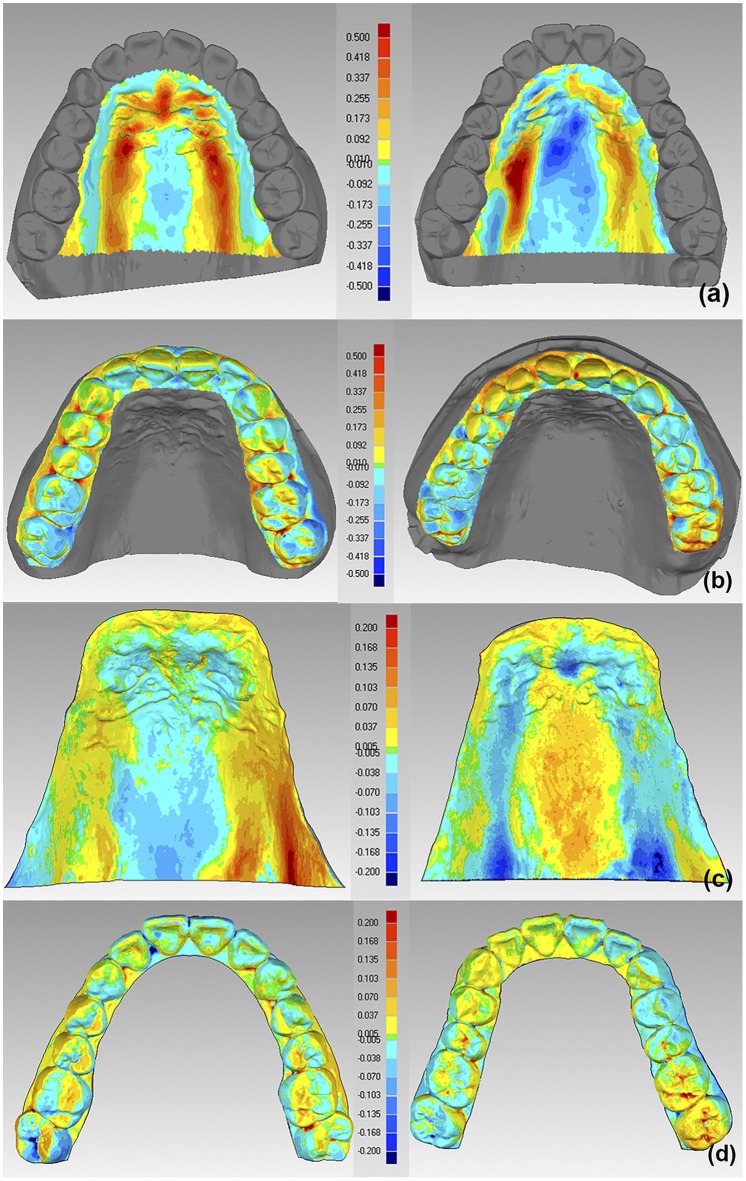
Color-coded deviation maps presentation after best fit alignment and 3D compare by Geomagic Qualify 12. (a) Trueness of digital impression for palatal soft tissues. Left: The top of the palatal vault was in the behind position. Right: The top of the palatal vault was in the front position. (b) Trueness of digital impression for full upper dentitions. (c) Precision of digital impression for palatal soft tissues. (d) Precision of digital impression for full upper dentitions. Color-coded scale unit: millimetre.

For 3D compare of palatal soft tissues between intraoral digital impressions and conventional impressions, it showed a mean deviation of (130.54±33.95)μm, of which the positive deviations was (185.84±51.11) μm and the negative deviation was (75.23±25.86)μm. The color-coded deviation map showed clear positive deviations at the palatal rugae and the two sides of the palatal vault, while negative deviations were found at other areas, especially at the midpalatal suture and the top of palatal vault region (Fig 4a). For 3D compare of full upper dentitions between intraoral digital impressions and conventional impressions, it showed a mean deviation of (80.01±17.78)μm, of which the positive deviation was (96.24±22.46)μm and the negative deviations was (63.78±16.18)μm. The color-coded deviation map showed positive deviations at the facial surfaces and gingivae of anterior teeth, the buccal and palatal gingivae of posterior teeth and occlusal surfaces of some posterior teeth. Negative deviations were observed at palatal surfaces of anterior teeth, occlusal surfaces of some posterior teeth and buccal gingivae of left posterior teeth (Fig 4b).

When comparing one digital impression with another of the same volunteer, it showed a precision of (55.26±11.21) μm for palatal soft tissues and (59.52±11.29)μm for full dentitions. The color-coded deviation map showed that positive and negative deviations were occurring alternatively and irregular deviations were visible across whole upper jaw (Fig 4c and 4d).

**Fig 5:**
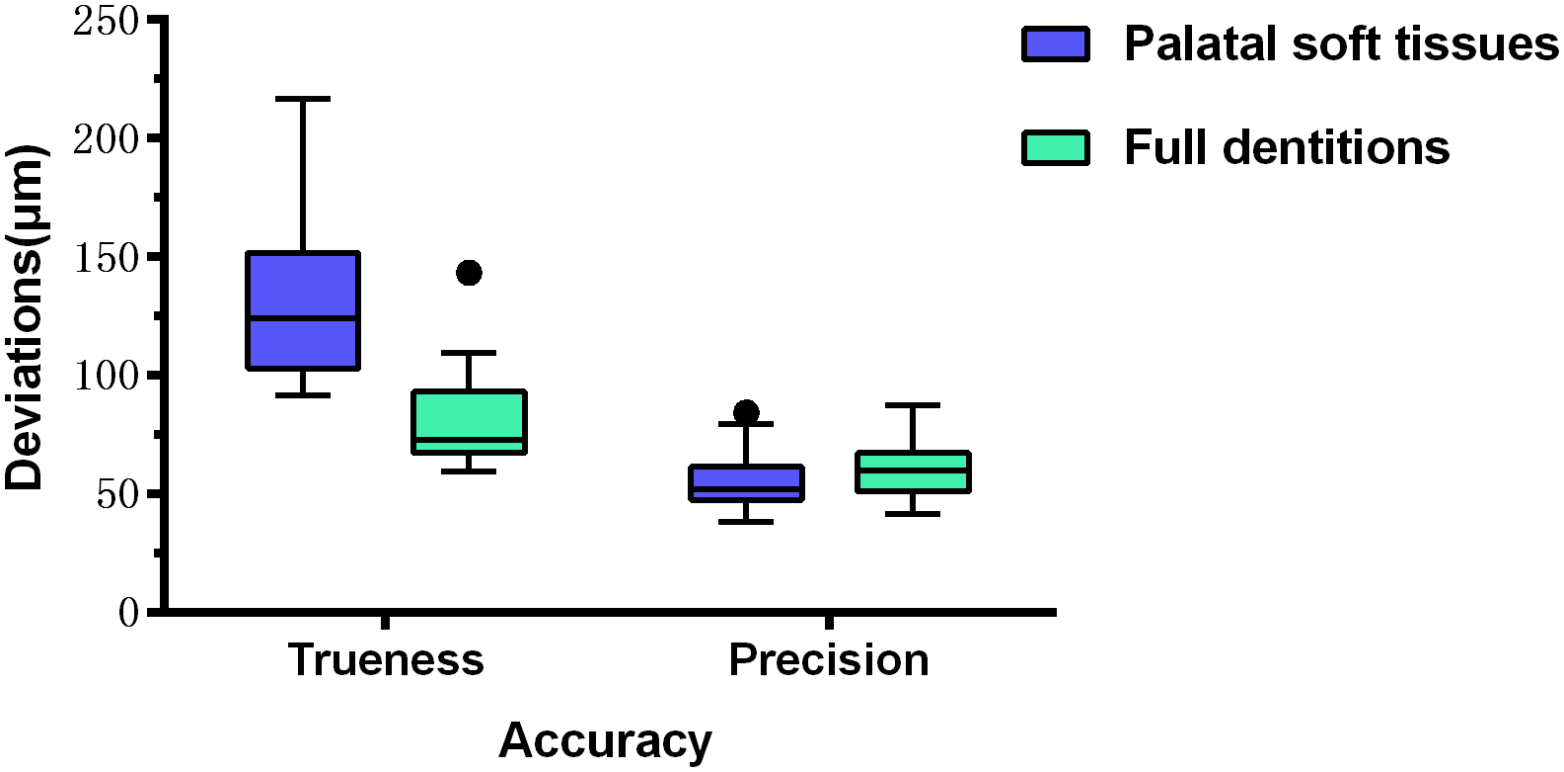
Box plot demonstrating accuracy measurement (trueness and precision) for palatal soft tissues and full upper dentitions. The black dots represent outliers.

It revealed a statistically significant difference compared the trueness of digital impressions for palatal soft tissues with that of full upper dentitions (p<0.001), while there was a slightly difference between the precision of digital impressions for palatal soft tissues and that for full upper dentitions (p = 0.049), (Fig 5).

### Effect of Arch Width on Accuracy of Digital Impressions

**Fig 6:**
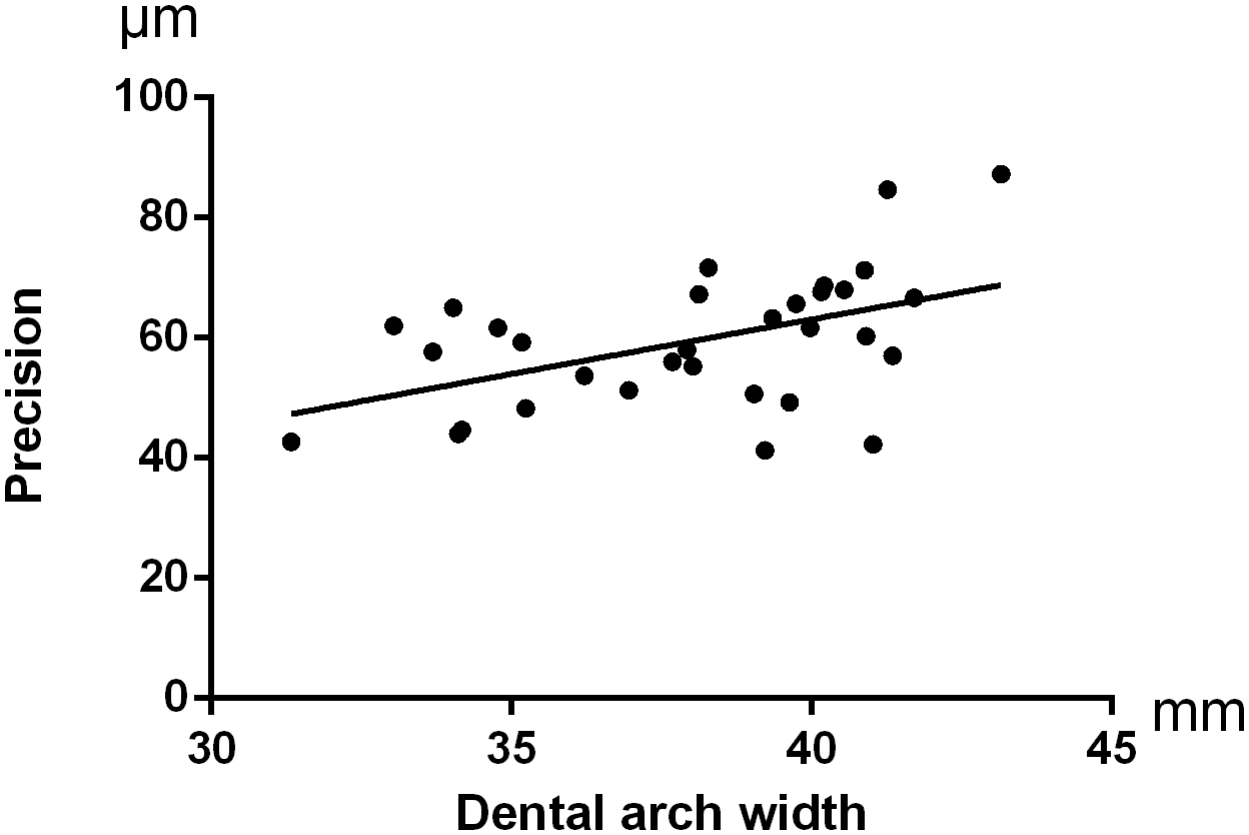
A linear correlation was found between arch width and precision of the digital impressions for full upper dentitions (r = 0.485,p = 0.002).

**Fig 7:**
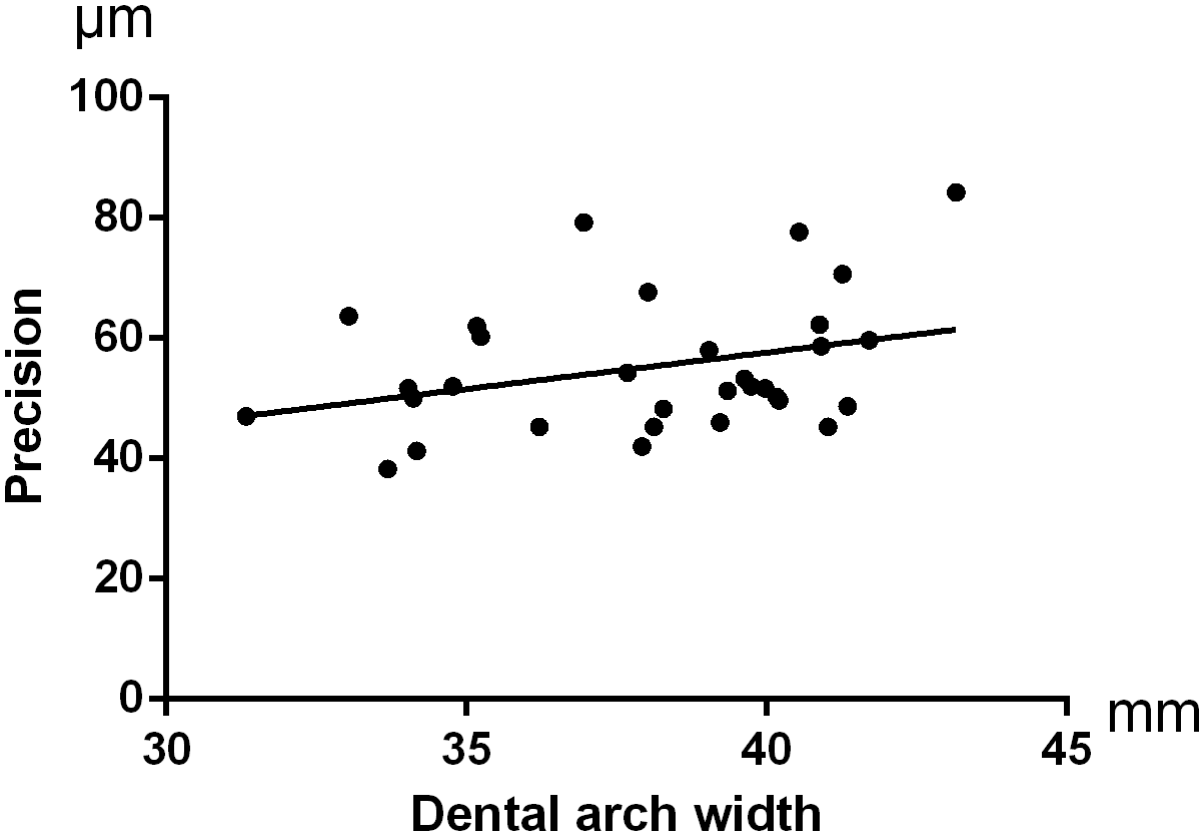
A linear correlation was found between arch width and precision of the digital impressions for palatal soft tissues (r = 0.326,p = 0.034).

No significant difference was presented when comparing accuracy of digital impressions for palatal soft tissues among the three different arch width groups (p = 0.380 for trueness and p = 0.147 for precision). Trueness of digital impressions for full dentitions was not influenced by arch width (p = 0.404), while precision of digital impressions for full dentitions was significantly influenced by arch width (p = 0.016), (Table 3). For further analysis, significant differences were observed in precision for full dentitions between Group Wide and Group Middle and Narrow, (p = 0.032 and p = 0.006, respectively). No significant differences were observed in precision for full dentitions between Group Middle and Group Narrow, (p = 0.395). The Pearson’s r-values showed a moderate linear correlation between arch width and precision of digital impressions for full upper dentitions (r = 0.485, p = 0.002) and a relatively weak linear correlation between arch width and precision of digital impressions for palatal soft tissues (r = 0.326, p = 0.034), (Figs 6 and 7).

**Table 3:** Effect of Arch Width on Mean(S.D.) Accuracy of Intraoral Digital Impressions for Palatal Soft Tissues and Full Dentitions in Millimeter.

Accuracy	Location	Group of arch width			Average	p-value
		Narrow	Medium	Wide		
**Trueness**	Palatal soft tissues	122.83(20.88)	126.64(39.19)	142.92(37.42)	130.54(33.95)	0.380
	Full upper dentition	74.22(13.52)	80.64(15.00)	85.05(23.77)	80.01(17.78)	0.404
**Precision**	Palatal soft tissues	51.17(8.65)	54.11(10.34)	60.73(13.18)	55.26(11.21)	0.147
	Full upper dentition	53.90(8.39)	57.64(8.71)	67.40(12.91)	59.52(11.29)*	0.016*

Asterisk(*): p < 0.05.

### Effect of Palatal Vault Height on Accuracy of Digital Impressions

No significant difference was presented when comparing accuracy of digital impressions for palatal soft tissues as well as full upper dentitions among the three different palatal vault height groups (palatal soft tissues: p = 0.747 for trueness and p = 0.961 for precision; full upper dentitions: p = 0.284 for trueness and p = 0.417 for precision), indicating that there was no effect of palatal vault height on accuracy of digital impressions (Table 4).

**Table 4:** Effect of Palatal Vault Height on Mean(S.D.) Accuracy of Intraoral Digital Impressions for Palatal Soft Tissues and Full Dentitions in Millimeter.

Accuracy	Location	Group of palatal vault height			Average	p-value
		Low	Medium	High		
**Trueness**	Palatal soft tissues	123.00(26.73)	133.74(40.07)	133.09(32.47)	130.54(33.95)	0.747
	Full upper dentition	73.58(12.28)	85.41(21.14)	78.05(15.68)	80.01(17.78)	0.284
**Precision**	Palatal soft tissues	55.19(13.53)	55.83(10.12)	54.44(11.68)	55.26(11.21)	0.961
	Full upper dentition	57.85(13.44)	57.83(9.69)	63.82(11.47)	59.52(11.29)	0.417

## Discussion

The null hypothesis was partly rejected. It was feasible to use the intraoral scanner to obtain digital impressions for whole upper jaws. Arch width could influence the precision of intraoral digital impressions, while palatal vault height might have no effect on the accuracy of intraoral digital impressions.

In present vivo study, it spent about (4mins58secs±1mins17secs) to finish the digital impression of whole upper jaw including interested soft and hard tissues, while the intraoral working time for conventional impression was at least 7 minutes 30 seconds according to manufacturer’s instruction. If we had taken the time for preparation and gypsum pouring into account, it would have been a more time-consuming procedure for conventional technique. It was also easier for operators to rescan and make up the faults occurred in the procedures of digital impression generation, while it might need to remake a new one from the beginning if there was something wrong with a conventional impression.

In terms of the trueness of digital impression in palatal areas, a total of (130.54±33.95)μm deviation was revealed. Color-coded deviation maps presented positive deviations at the palatal rugae as well as the two sides of the palatal vault. The clear positive deviations implied that the differences between the digital impression and conventional impression were derived from the flexibility of palatal mucosa mainly. Intraoral digital scan has no touch with the mouth structure. But vinyl polysiloxane materials were pressed onto volunteer’s upper jaw, and oral mucosa at the palatal rugae and the two sides of the palatal vault would be oppressed certainly. The pressed force will inevitably different from the operator’s two hands and habits. So the digital impression was “over” the conventional impression at these regions. Due to the individual differences and position diversity, the flexibility of palatal mucosa was so different that it was colorful in color-coded deviation maps, ranging from yellow to red representing different levels of deviations. On the other hand, negative deviations were observed at other areas and especially darker deviation was showed at the midpalatal suture and the top of palatal vault region. The phenomenon was partially in agreement with the in vitro result revealed by Patzelt S.B.M. et al[15]. They found the mean trueness of digitizing edentulous jaws ranged from 44.1–591.8μm and the accuracy of the scanners differs significantly. Their visualization of the data sets showed greater deviations presented in dark blue (up to 700 μm) in the palatal areas. The reason could attribute to: (1) a smooth surface and poorly traceable structures in the middle of palatal areas which could cause faults in data process; (2) scan wand operated in free-floating position without sufficient supporting structures and hands shake during the scan procedure[15]. In terms of the process of making a conventional impression in the present study, the midpalatal suture and the top of palatal vault region were much far away from the press area and less pressure were applied, which may not cause mucosa much deformation. So the intraoral digital impression was “under” the reference in these areas and the negative deviations were shown in color-coded deviation maps.

For the part of the trueness of digital impression for full dentitions, it showed a mean deviation of (80.01±17.78)μm. Positive deviations were observed at the facial surfaces and gingivae of anterior teeth, the buccal and palatal gingivae of posterior teeth and occlusal surfaces of some posterior teeth. The smooth surface of anterior teeth and steep-angled at incisal edges might be the explanation of clear deviations at the facial surfaces and gingivae of anterior teeth[9]. Lee S. found the volumetric deviations of fossae areas were statistically different between the gypsum and digitally milled models. Such deviations might derived from the limited ability of the scanner[5]. The present study also found positive deviations (red deviations) at some interproximate spaces and fossae on the posterior teeth, which indicated that there were remaining saliva at these areas. Another explanation was that scanning light had more difficulties to arrive at these deep areas then catch the morphology of them. To improve these situations, it should be recommended that saliva on teeth surface needed to be removed more often during the scanning procedure, also that multiple and slow scans from different directions were necessary in order to compensate for shadowing effects in deep and narrow areas[10].

The present precision study used the method as Patzelt S.B.M. that digital impressions were compared with each other, so the reference model differed in every comparison pairs[15]. The color-coded deviation maps in this present study shown positive and negative deviations occurred alternatively across the entire dental arch, which was in accordance with the results of Ender A.[20]. The mean value of precision for full dentitions was (59.52±11.29)μm in this vivo study. Similarly, a previous study declared a precision of 50μm for intraoral scanning by intraoral scanner and a precision of 25μm for extraoral scanning by the same intraoral scanner[9]. In the present study, precision of digital impressions for full upper dentition decreased with the increased arch width, furthermore, there was a moderate linear correlation between precision and arch width. This observation supported findings previously reported by Su T.S. et al[22], who did an in vitro study and stated precision of intraoral digital impression with TRIOS decreased with the increased scanning scope. Mehl A. et al also found less accuracy for quadrant images compared to single tooth images using CEREC Bluecam intraoral scanner[23]. Combined with the present study, we may consider that more precise errors of intraoral digital impression would happen during quadrant scanning than single tooth scanning, and the larger the scanning region the less repeatability might occur.

The mean value of precision for palatal soft tissues was (55.26±11.21)μm. The digital impressions for palatal soft tissues showed a little better precision than the digital impression for full upper dentitions. The difference between them was significant but at the edge of statistical significance. Rudolph H. indicated that the tooth shape was the dominant factor for precision. Qualitative analysis revealed the largest deviations in the areas of strong changes of curvature[24]. The surface of teeth was much more complicated than palatal mucosa, which could be the reason to lead to more precision error. Although a relatively weak linear correlation was found between arch width and precision of intraoral digital impressions for palatal soft tissues (r = 0.326, p = 0.034), the effect of arch width on the accuracy of digital impressions for palatal soft tissues was not such significant according to the results of the present study.

Palatal vault height had no effect on accuracy of intraoral digital impressions in this study. However, it could be seen that darker and more obvious blue deviations were presented in figures of those volunteers with narrower and higher palatal vault. We began this in vivo study at the beginning of 2015 when the standard scanning head was the only choice. The standard scanning head was larger than the space of the top of narrower palatal vault, so the scanning head was far from mucosa surface at some areas and was not able to parallel to these mucosa surface. Now new TRIOS 3 with smaller scanning head has been introduced to the market, and it will make possible to capture the images of the top of palate with better quality. Actually, it was more complicated to explain the influencing factors on the accuracy of palatal digital impressions for the reason of the individual differences in the shape of palatal vaults and flexibility of palatal mucosa. Thus it may need to expand the sample size and scan the tissues by smaller scanning head for further analysis.

In addition, the reference datasets derived from conventional impressions represented volunteers’ oral conditions that oral mucosa was oppressed during impression procedure. The color-coded deviation maps in this study only gave a preliminary and direct viewing of difference between digital impressions and conventional impressions in the regions of palatal soft tissues. This difference is definite, yet whether it is clinically acceptable is still unsure. So our further clinical studies will design and fabricate the RPD prostheses with these datasets and the adaptation of dental prostheses will be measured in mouth to evaluate the accuracy of intraoral digital impression.

This is one of the first studies that focus on the accuracy of digital impressions for both palatal soft tissues and full dentitions respectively. Volunteers with implants or carries were all excluded to avoid the errors. Only one volunteer involved in the study was a light smoker with good oral hygiene so was not separated from others in statistical analysis. However, smoking could lead to the increase of the keratinized and parakeratinized tissues then there might be a difference in keratization degree of soft tissues between heavy-smokers and non-smokers. More studies are necessary in these areas.

## Conclusions

Within the limitation of this study, it was feasible to use the intraoral scanner to obtain digital impressions for whole upper jaws. Trueness of digital impressions for full dentitions was better than that for palatal soft tissues. Precision of digital impressions for palatal soft tissues was a little better than that for full dentitions. Arch width could influence the precision of intraoral digital impressions, while palatal vault height might have no effect. It should be confirmed in further studies that whether the accuracy of digital impressions for whole jaws is clinically acceptable.

## References

[1] Seelbach P, Brueckel C, Wostmann B. Accuracy of digital and conventional impression techniques and workflow. Clin Oral Investig. 2013;17(7):1759–64. 10.1007/s00784-012-0864-4 23086333

[2] Syrek A, Reich G, Ranftl D, Klein C, Cerny B, Brodesser J. Clinical evaluation of all-ceramic crowns fabricated from intraoral digital impressions based on the principle of active wavefront sampling. J Dent. 2010;38(7):553–9. 10.1016/j.jdent.2010.03.015 20381576

[3] Brawek PK, Wolfart S, Endres L, Kirsten A, Reich S. The clinical accuracy of single crowns exclusively fabricated by digital workflow—the comparison of two systems. Clin Oral Investig. 2013;17(9):2119–25. 10.1007/s00784-013-0923-5 23371756

[4] Moreno A, Gimenez B, Ozcan M, Pradies G. A clinical protocol for intraoral digital impression of screw-retained CAD/CAM framework on multiple implants based on wavefront sampling technology. Implant dentistry. 2013;22(4):320–5. 10.1097/ID.0b013e3182980fe9 23817542

[5] Lee SJ, Betensky RA, Gianneschi GE, Gallucci GO. Accuracy of digital versus conventional implant impressions. Clin Oral Implants Res. 2015;26(6):715–9. 10.1111/clr.12375 24720423 PMC4428303

[6] Goodacre CJ, Garbacea A, Naylor WP, Daher T, Marchack CB, Lowry J. CAD/CAM fabricated complete dentures: concepts and clinical methods of obtaining required morphological data. The Journal of prosthetic dentistry. 2012;107(1):34–46. 10.1016/S0022-3913(12)60015-8 22230914

[7] Jiao T, Zhu C, Dong X, Gu X. Rehabilitation of maxillectomy defects with obturator prostheses fabricated using computer-aided design and rapid prototyping: a pilot study. Int J Prosthodont. 2014;27(5):480–6. 10.11607/ijp.3733 25191895

[8] The official website of 3 Shape Corporation. Available: http://www.3shape.com/en/new+products/ dental+labs/lab+scanners.

[9] Flugge TV, Schlager S, Nelson K, Nahles S, Metzger MC. Precision of intraoral digital dental impressions with iTero and extraoral digitization with the iTero and a model scanner. Am J Orthod Dentofacial Orthop. 2013;144(3):471–8. 10.1016/j.ajodo.2013.04.017 23992820

[10] Quaas S, Rudolph H, Luthardt RG. Direct mechanical data acquisition of dental impressions for the manufacturing of CAD/CAM restorations. Journal of dentistry. 2007;35(12):903–8. 17980951 10.1016/j.jdent.2007.08.008

[11] Svanborg P, Skjerven H, Carlsson P, Eliasson A, Karlsson S, Ortorp A. Marginal and internal fit of cobalt-chromium fixed dental prostheses generated from digital and conventional impressions. Int J Dent. 2014;2014:534382 10.1155/2014/534382 24723954 PMC3958727

[12] Almeida e Silva JS, Erdelt K, Edelhoff D, Araujo E, Stimmelmayr M, Vieira LC, et al Marginal and internal fit of four-unit zirconia fixed dental prostheses based on digital and conventional impression techniques. Clin Oral Investig. 2014;18(2):515–23. 10.1007/s00784-013-0987-2 23716064

[13] Berrendero S, Salido MP, Valverde A, Ferreiroa A, Pradies G. Influence of conventional and digital intraoral impressions on the fit of CAD/CAM-fabricated all-ceramic crowns. Clin Oral Investig. 2016. 10.1007/s00784-016-1714-6 26800669

[14] Gjelvold B, Chrcanovic BR, Korduner EK, Collin-Bagewitz I, Kisch J. Intraoral Digital Impression Technique Compared to Conventional Impression Technique. A Randomized Clinical Trial. J Prosthodont. 2015. 10.1111/jopr.12410 26618259

[15] Patzelt SB, Vonau S, Stampf S, Att W. Assessing the feasibility and accuracy of digitizing edentulous jaws. Journal of the American Dental Association. 2013;144(8):914–920. 23904578 10.14219/jada.archive.2013.0209

[16] Kattadiyil MT, Mursic Z, AlRumaih H, Goodacre CJ. Intraoral scanning of hard and soft tissues for partial removable dental prosthesis fabrication. J Prosthet Dent. 2014;112(3):444–8. 10.1016/j.prosdent.2014.03.022 24882595

[17] Michalakis KX, Asar NV, Kapsampeli V, Magkavali-Trikka P, Pissiotis AL, Hirayama H. Delayed linear dimensional changes of five high strength gypsum products used for the fabrication of definitive casts. The Journal of prosthetic dentistry. 2012;108(3):189–95. 10.1016/S0022-3913(12)60146-2 22944315

[18] Takanashi T, Shimamura I, Sakurai K. Influence of width and depth of palatal vault on rigidity of palatal strap: a finite element study. J Prosthodont Res. 2009;53(2):95–100. 10.1016/j.jpor.2008.10.002 19318080

[19] DIN ISO 5725–3:1994. Accuracy (trueness and precision) of measurement methods and results Part 3-Intermediate measures of the precision of a standard measurement(ISO 5725–3:1994).

[20] Ender A, Mehl A. Accuracy of complete-arch dental impressions: A new method of measuring trueness and precision. The Journal of Prosthetic Dentistry. 2013;109(2):121–8. 10.1016/S0022-3913(13)60028-1 23395338

[21] Yan H, Xu YY, Zhao NQ. Medical Statistics, People’s Medical Publishing House. 2005;Chapter 16, pp.258, ISBN:7117069082.

[22] Su TS, Sun J. Comparison of repeatability between intraoral digital scanner and extraoral digital scanner: An in-vitro study. Journal of prosthodontic research. 2015. 10.1016/j.jpor.2015.06.002 26211702

[23] Mehl A, Ender A, Mörmann W; Attin T. Accuracy testing of a new intraoral 3d camera. International journal of computerized dentistry. 2009;12(1):11–28. 19213357

[24] Rudolph H, Luthardt RG, Walter MH. Computer-aided analysis of the influence of digitizing and surfacing on the accuracy in dental CAD/CAM technology. Computers in Biology and Medicine. 2007;37(5):579–87. 16844107 10.1016/j.compbiomed.2006.05.006

